# High-throughput genotyping of a full voltage-gated sodium channel gene via genomic DNA using target capture sequencing and analytical pipeline MoNaS to discover novel insecticide resistance mutations

**DOI:** 10.1371/journal.pntd.0007818

**Published:** 2019-11-18

**Authors:** Kentaro Itokawa, Tsuyoshi Sekizuka, Yoshihide Maekawa, Koji Yatsu, Osamu Komagata, Masaaki Sugiura, Tomonori Sasaki, Takashi Tomita, Makoto Kuroda, Kyoko Sawabe, Shinji Kasai

**Affiliations:** 1 Pathogen Genomics Center, National Institute of Infectious Diseases, Tokyo, Japan; 2 Department of Medical Entomology, National Institute of Infectious Diseases, Tokyo, Japan; 3 Antimicrobial Resistance Research Center, National Institute of Infectious Diseases, Tokyo, Japan; 4 Global Research and Development Department, Fumakilla Limited, Hiroshima, Japan; 5 Research and Development Department, Fumakilla Limited, Hiroshima, Japan; University of Queensland, AUSTRALIA

## Abstract

In insects, the voltage-gated sodium channel (VGSC) is the primary target site of pyrethroid insecticides. Various amino acid substitutions in the VGSC protein, which are selected under insecticide pressure, are known to confer insecticide resistance. In the genome, the *VGSC* gene consists of more than 30 exons sparsely distributed across a large genomic region, which often exceeds 100 kbp. Due to this complex genomic structure, it is often challenging to genotype full coding nucleotide sequences (CDSs) of *VGSC* from individual genomic DNA (gDNA). In this study, we designed biotinylated oligonucleotide probes from CDSs of *VGSC* of Asian tiger mosquito, *Aedes albopictus*. The probe set effectively concentrated (>80,000-fold) all targeted regions of gene *VGSC* from pooled barcoded Illumina libraries each constructed from individual *A*. *albopictus* gDNAs. The probe set also captured all orthologous *VGSC* CDSs, except some tiny exons, from the gDNA of other Culicinae mosquitos, *A*. *aegypti* and *Culex pipiens* complex, with comparable efficiency as a result of the high nucleotide-level conservation of *VGSC*. To improve efficiency of the downstream bioinformatic process, we developed an automated pipeline—MoNaS (Mosquito Na^+^ channel mutation Search)—which calls amino acid substitutions in the *VGSC* from NGS reads and compares those to known resistance mutations. The proposed method and our bioinformatic tool should facilitate the discovery of novel amino acid variants conferring insecticide resistance on VGSC and population genetic studies on resistance alleles (with respect to the origin, selection, and migration etc.) in both clinically and agriculturally important insect pests.

## Author summary

The Voltage Gated Sodium Channel (VGSC) in insect is targeted by pyrethroid insecticides and genetic variations in the protein are known to confer pyrethroid resistance. Since the *VGSC* gene in genome consists of many exons and long introns, there is no simple method to genotype whole of coding regions from the genomic DNA of insect. Here, we designed hybridization capture probe set to concentrate *VGSC* coding exons in NGS library from individual genomic DNA of the arbovirus vector mosquito *Aedes albopictus*. The probe set we designed was able to capture *VGSC* exons not only from *A*. *albopictus* genomic DNA but also from genomic DNA of two other mosquito species belonging to the same subfamily only with slight decrease of efficiency. The technology will allow unbiased analysis of the *VGSC* gene in multiple mosquito species with relatively low sequencing cost and enhance discovery of new resistance mutations.

## Introduction

Medically important insect vectors such as mosquitoes undergo strong selective pressure from insecticides in the field where control of vector-borne diseases such as malaria, dengue and zika is applied. This pressure often results in development of resistance against insecticides, which pose a potential risk for public health [1,2]. Although there can be various mechanisms by which insects acquire resistance against insecticides, two main physiological mechanisms are well known. One is enhanced metabolism of insecticide active ingredients by detoxification enzymes including cytochrome P450s, glutathione S-transferases and carboxyl esterases. The other mechanism is target-site insensitivity due to a point mutation(s) in the insecticide’s target-protein which reduces the interaction between the two molecules.

Synthetic pyrethroids are the most frequently used insecticide group for the control of clinically important mosquitos. The mode of pyrethroid’s toxicity is inhibition of the voltage-gated sodium channel (VGSC) in the nervous system [3]. Development of resistance against pyrethroids, which is known as knockdown resistance (*kdr*), was first reported in housefly, *Musca domestica*, in 1950s [4]. The *kdr* phenotype as well as another distinct phenotype, super-*kdr*, was eventually linked to amino acid (aa) substitutions on the two positions, L1014F and L1014F+M918T, respectively, on the gene coding VGSC protein [5,6]. Currently, these and other aa substitutions have been found to be associated with resistance in many medical and agricultural insect pests [7,8]. With this historical background, aa substitutions in a variety of insect species are often described with projection to the corresponding aa position in *M*. *domestica* VGSC for comparison. The VGSC is highly conserved among insects, and many of the resistance-conferring aa substitutions are seen in different species [9]. Therefore, it is relatively straightforward to infer the effect of certain aa substitutions in any species if the effect of those substitutions has already been elucidated in other species. Analyzing nucleotide sequences of an entire coding sequence (CDS) of the *VGSC* gene from genomic DNA (gDNA), however, is complicated because *VGSC* genes typically consist of many (>30) small exons sparsely distributed across a large genomic region which often exceeds 100 kbp. Therefore, most studies employing polymerase chain reaction (PCR) and direct sequencing usually cover only restricted regions where the known resistance-conferring substitutions are frequently found; e.g., IIS5–6 [7,8]. Such a bias may lower the chance of discovering novel resistance mutations existing outside the region investigated.

*Aedes albopictus*, the Asian tiger mosquito, is a medically important mosquito species ubiquitously present on most continents on the Earth. In some regions where the other effective vector, *A*. *aegypti*, is absent, the species often take a main role for transmitting chikungunya and dengue viruses [10,11]. The *kdr* substitution in *A*. *albopictus* had not been reported until the F1534C allele was discovered in Singapore 2009 [12]. Since this discovery, F1534C and other *kdr* substitutions at the same aa position, F1534S and F1534L, were reported from in *A*. *albopictus* in various geographic locations worldwide [13–15]. More recently, we also discovered the new *kdr* substitution V1016G in *A*. *albopictus* by extending the region of the search for mutations [16].

Next-generation sequencing (NGS) technology has reduced the cost and time of DNA sequencing by orders of magnitude. The recent *Anopheles gambiae* 1000 Genomes project, Ag1000G, has uncovered a number of previously unknown nonsynonymous mutations in the *VGSC* gene in *A*. *gambiae* and *A*. *coluzzii* [17], some of which have been suspected to cause resistance directly or indirectly. Although whole-genome sequencing (WGS) may discover novel variants of *VGSC* unequivocally, this naive approach is still too costly per sample just for analyzing *VGSC*. Alternatively, we considered an enrichment approach involving hybridization of oligo DNA/RNA [18] which is often employed to selectively sequence targeted genomic regions for studies e.g. on genotyping of disease-related genes in humans. This technology is aimed at increasing the depth of reads and the number of samples to be multiplexed per given sequencing capacity in return for limiting the region to be analyzed. In this study, we designed biotinylated oligonucleotide DNA probes from *A*. *albopictus VGSC* CDSs. The probe set efficiently concentrated targeted regions from the gDNA of individual *A*. *albopictus*. Although the probe set was designed from the *A*. *albopictus VGSC* gene, the same probe set captured most CDSs of other important arbovirus vectors *A*. *aegypti* and *Culex pipiens* complex, in which several *kdr* mutations are already known to exist [8,19], as a result of the high nucleotide conservation of *VGSC*. This technology allows for full-CDS analysis of the complex *VGSC* gene in a relatively low-cost and highly multiplexed manner, which is expected to promote discoveries of novel resistance-conferring aa substitutions both in medical and agricultural insect pests.

## Results

From the *A*. *albopictus* genome assembly AaloF1 [20], 229 oligo DNA probes (xGen Custom Target Capture Probes, IDT) were designed. Of these, 145 probes target *VGSC* coding regions from gene model refined in this study (see Material & Methods). The rest of probes target exons of other genes located on the same scaffold as *VGSC*, JXUM01S000562, to detect signal of positive selection by method such as Sabeti, et al [19]. However, in the more recent contiguous assembly of the C6/36 cell line [21], many of those genes do not locate in proximity to *VGSC*, which suggests possible misassembly in either of the two assemblies. Therefore, in this study, we only evaluate performance of capture for *VGSC* gene. See Material & Methods for more details.

**Fig 1:**
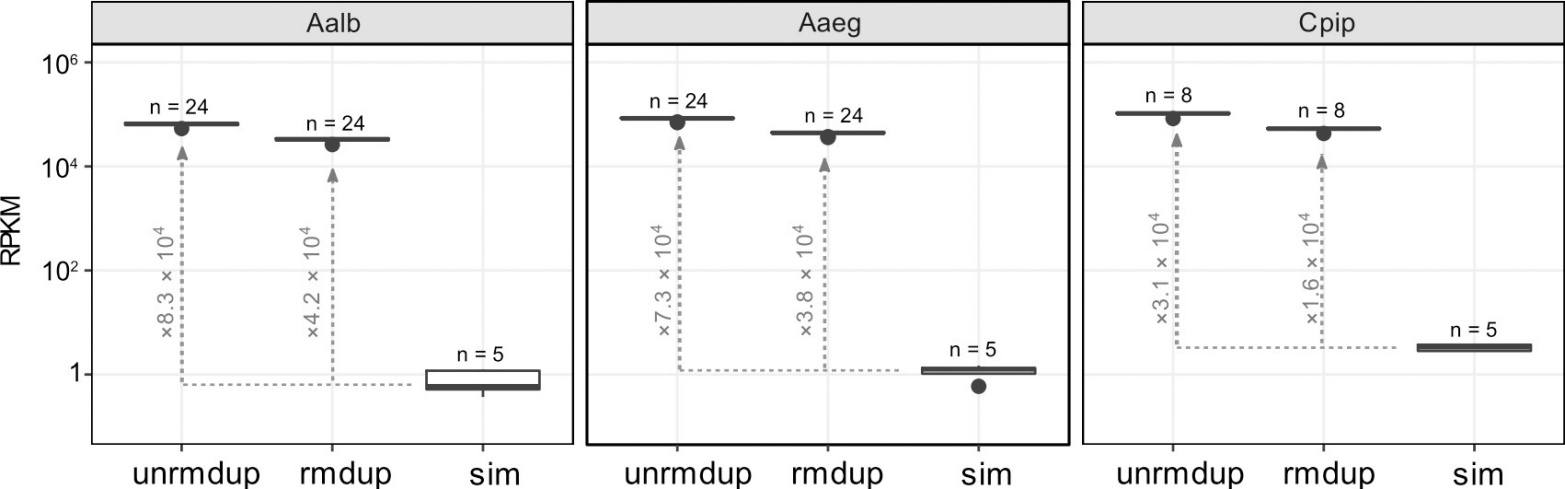
The NGS reads are enriched in targeted *VGSC* exons by capture Distributions of RPKM (number of sequencing reads overlapping to the targeted *VGSC* exons per 1 kbp total exon length and one million reads) for *A*. *albopictus* (Aalb), *A*. *aegypti* (Aaeg) and *C*. *pipiens* complex (Cpip). Labels “unrmdup” and “rmdup” indicate before and after removal of PCR duplicates, respectively. Label “sim” indicates simulated whole genome shotgun (WGS) reads randomly drawn from genome of each species (replicated five times in each species). The values associated with dotted line with arrowhead indicate sizes of fold-change (levels of enrichment) compared to simulated WGS data.

Targeted sequencing of the *VGSC* gene was conducted for 56 mosquito gDNA samples including *A*. *albopictus*, *A*. *aegypti* and *Culex pipiens* complex subspecies (*C*. *quinquefasciatus*, *C*. *pipiens pallens* and *C*. *pipiens* form *molestus*) (Table 1) in a single run of Illumina MiniSeq. Each individual gDNA was indexed with different barcoded adapters, and in total, 4.9 million read pairs (150 bp PE) were assigned to the 56 samples. From those samples, 40–170, 50–120, and 63–100 K read-pairs were obtained from each individual mosquito of *A*. *albopictus* and *A*. *aegypti* and *C*. *pipiens* complex, respectively. The raw read data were deposited to DDBJ Sequence Read Archive (DRA) under BioProjectID: PRJDB7889 and each accession numbers as listed in Table 1. For comparison, random sequence read sets derived from reference genome sequences as a simulated output of WGS approach were mapped in same manner to the captured library data. In *A*. *albopictus*, 44% of all reads on average overlapped with the *VGSC* CDSs targeted (Reads overlapping per kilobase exon and per million sequenced reads: RPKM = 6.5 × 10^4^), which was approximately 8.3 × 10^4^-fold enrichment compared to the simulated whole-genome shotgun sequencing (Fig 1). The efficiency reduced to 4.2 × 10^4^-fold enrichment after PCR duplicates were removed. Although the probe set was designed based on only the *A*. *albopictus* genomic sequence, the same probe set captured *VGSC* CDSs from the gDNA of other mosquitos, *A*. *aegypti* and *C*. *pipiens* complex, at a similar on-target rate to *A*. *albopictus* (Fig 1).

**Table 1:** Mosquito samples used in this study.

ID	Species	Lab. Colony / Wild	num	Description	DDBJ Accession no.
Aalb-SP	*A*. *albopictus*	Lab. Colony	8	Originated from Singapore in 2016. This strain (SP16C) is known to possess 1534C *kdr* variant [16].	DRR167925–932
Aalb-Viet	*A*. *albopictus*	Lab. Colony	2	Originated from Hanoi, Viet Nam in 2016.	DRR167935–936
Aalb-Okayama	*A*. *albopictus*	Lab. Colony	2	Originated from Okayama, Japan in 2015.	DRR167923–924
Aalb-Toyama	*A*. *albopictus*	Lab. Colony	2	Originated from Tokyo, Japan in 2015.	DRR167933–934
Aalb-Ishigaki	*A*. *albopictus*	Lab. Colony	2	Originated from Ishigaki-zima, Okinawa, Japan in 2016.	DRR167921–922
Aalb-Yona	*A*. *albopictus*	Wild	8	Caught wild from Yonaguni-zima, Okinawa, Japan in 2017.	DRR167937–944
Aaeg-Mex	*A*. *aegypti*	Lab. Colony	16	Originated from Monterrey, Mexico in 2008.	DRR167897–912
Aaeg-SP	*A*. *aegypti*	Lab. Colony	8	Originated from Singapore in 2009. This strain is known to possesses 989P–1016G kdr haplotype [23].	DRR167913–920
Cpip-JNA	*C*. *quinquefasciatus*	Lab. Colony	2	This strain was selected for *CYP9M10* genotype [28] from JHB strain originated from Johannesburg, South Africa in 2001 [29].	DRR167945–946
Cpip-JPP	*C*. *quinquefasciatus*	Lab. Colony	2	Originated from Saudi Arabia, selected by permethrin for 20 generations [30]. This strain is known to possesses 1014F *kdr* variant [24].	DRR167947–948
Cpip-Ryo	*C*. *pipiens pallens*	Lab. Colony	2	Originated from Kanagawa, Japan in 2015.	DRR167951–952
Cpip-JP_mix	*C*. *pipiens* form *molestus*	Lab. Colony	2	Mixed from several lab. colonies originated from different places of Japan in 2003–2004.	DRR167949–950

**Fig 2:**
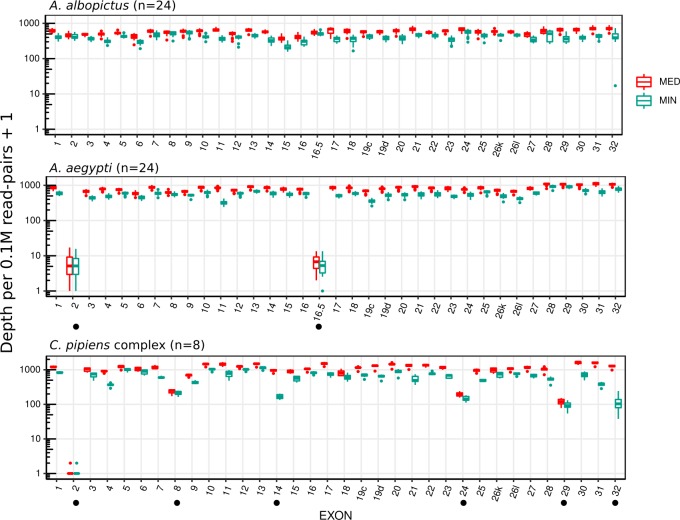
Coverage of targeted *VGSC* exons Distribution of median (MED) and minimum (MIN) depths per nucleotide (on a logarithmic scale) within each exon and each individual sample after PCR duplicates were removed. Exons labeled with “●” contained nucleotide sites with relatively low coverage.

Fig 2 shows a distribution of median and minimum sequencing depths normalized to total amount of sequencing effort within each CDS in each individual sample after PCR duplicates were removed. In *A*. *albopictus*, most nucleotides in all exons were covered deeply with minimum bias in all samples. In *A*. *aegypti* and *C*. *quinquefasciatus*, however, some exons were covered at relatively low depth partly or entirely. In particular, exons 2 and 16.5 were covered at nearly or absolutely zero depth.

**Fig 3:**
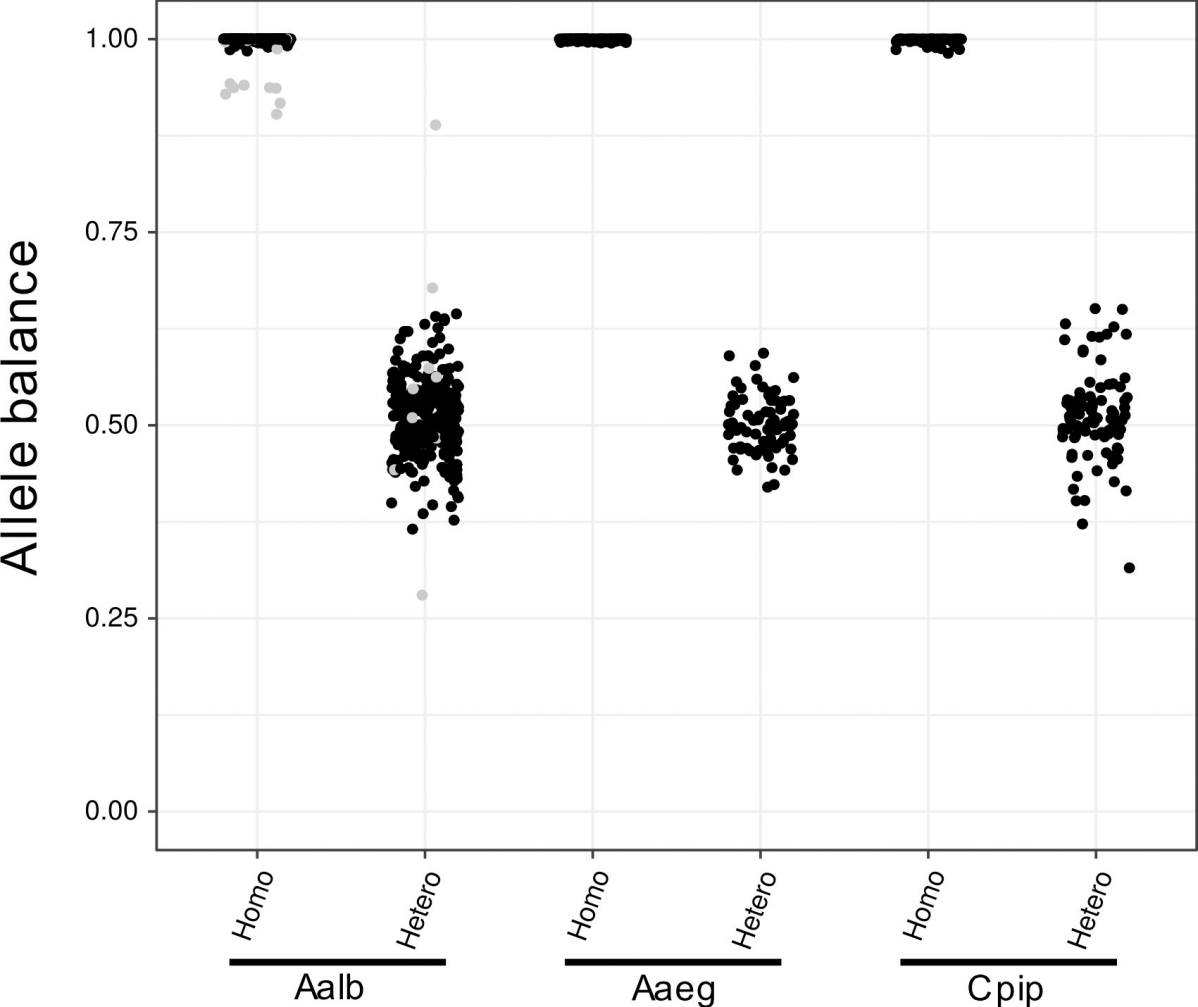
The allele balance in genotypes containing a variant The distribution of allele balance in read depth for each allele at heterozygous or homozygous genotypes containing alternative allele(s). The balance was calculated as [read depth of the first allele in “GT” info] / [total depth] in the VCF format. Gray points in *A*. *albopictus* are at the genotype at GGT (Gly) trinucleotide repeats in exon32.

Fig 3 presents a distribution of the allele balance in genotypes containing single or multiple nucleotide variants (SNVs or MNVs) called by FreeBayes [22]. The ratio of the first allele in a heterozygous genotype was distributed mostly around 50%, which was substantially different from the homozygous genotype (near 100%) except for one SNV or MNV site in exon 32 of the *A*. *albopictus* gene located in the GGT (Gly) trinucleotide tandem repeats variable in length near the C-terminus of VGSC, where accurate calling of the genotype is difficult.

Aa substitutions identified in the samples used in this study are listed in Table 2. Of those, all previously known fixed aa substitutions, F1534C and A2023T in Aalb-SP [16], S989P and V1016G in Aaeg-SP [23], and L1014F in Cpip-JPP [24], were recalled correctly. In the Aaeg-Mex strain, other previously know *kdr* mutations, V410L, V1016I, and F1534C [25–27], were detected. Other aa substitutions—C749*Y (*in mosquito aa coordinates because there was no corresponding aa in *M*. *domestica* reference), A2023T and G2046E in *A*. *albopictus*, S723T in *A*. *aegypti*, and K109R, Y319F, T1632S, E1633D, E1856D, G2051A, and A2055V in *C*. *pipiens* complex—are not known to their effects on insecticide susceptibility.

**Table 2:** Detected amino-acid substitutions.

Species	Population	n	Amino-acid substitutions (num. of homozygous, heterozygous individuals)
*A*. *albopictus*	Aalb-SP	8	F1534C**(8,0); A2023T(8,0)
	Aalb-Viet	2	A2023T(0,1)
	Aalb-Okayama	2	C749*Y(0,1); A2023T(0,1); G2046E(0,1)
	Aalb-Toyama	2	A2023T(0,1)
	Aalb-Ishigaki	2	A2023T(1,0)
	Aalb-Yona	8	A2023T(0,2)
*A*. *aegypti*	Aaeg-Mex	16	V410L**(1,7); S723T(1,7); V1016I**(1,7); F1534C**(16,0)
	Aaeg-SP	8	S989P**(8,0); V1016G**(8,0)
*C*. *quinquefasciatus*	Cpip-JNA	2	K109R(0,1); T1632S(0,1); E1633D(0,1); G2051A(0,1); A2055V(0,1)
	Cpip-JPP	2	R261K(2,0); L1014F**(2,0)
*C*. *pipiens pallens*	Cpip-Ryo	2	Y319F(2,0); T1632S(0,2); E1633D(0,2)
*C*. *pipiens* form *molestus*	Cpip-JP_mix	2	Y319F(2,0); L1014F**(2,0); T1632S(2,0); T1633D(2,0); E1856D(2,0)

The amino-acid coordination is *M*. *domestica* except C749Y with asterisk (*) since there was no corresponding amino-acid in *M*. *domestica* (genbank id: AAB47604). Double asterisks (**) indicate known *kdr* substitutions conferring pyrethroid resistance. Variants seen on the Gly repeats near the C-terminal are omitted.

## Discussion

In this study, we evaluated the potential of targeted enrichment sequencing technology to genotype mosquito *VGSC* CDSs from individual gDNA samples. The result of the experiment is quite promising, most nucleotides of *VGSC* CDSs were covered at sufficient read depths even in samples with less than a 30 Mbp (0.1 million read-pairs) sequencing effort.

**Fig 4:**
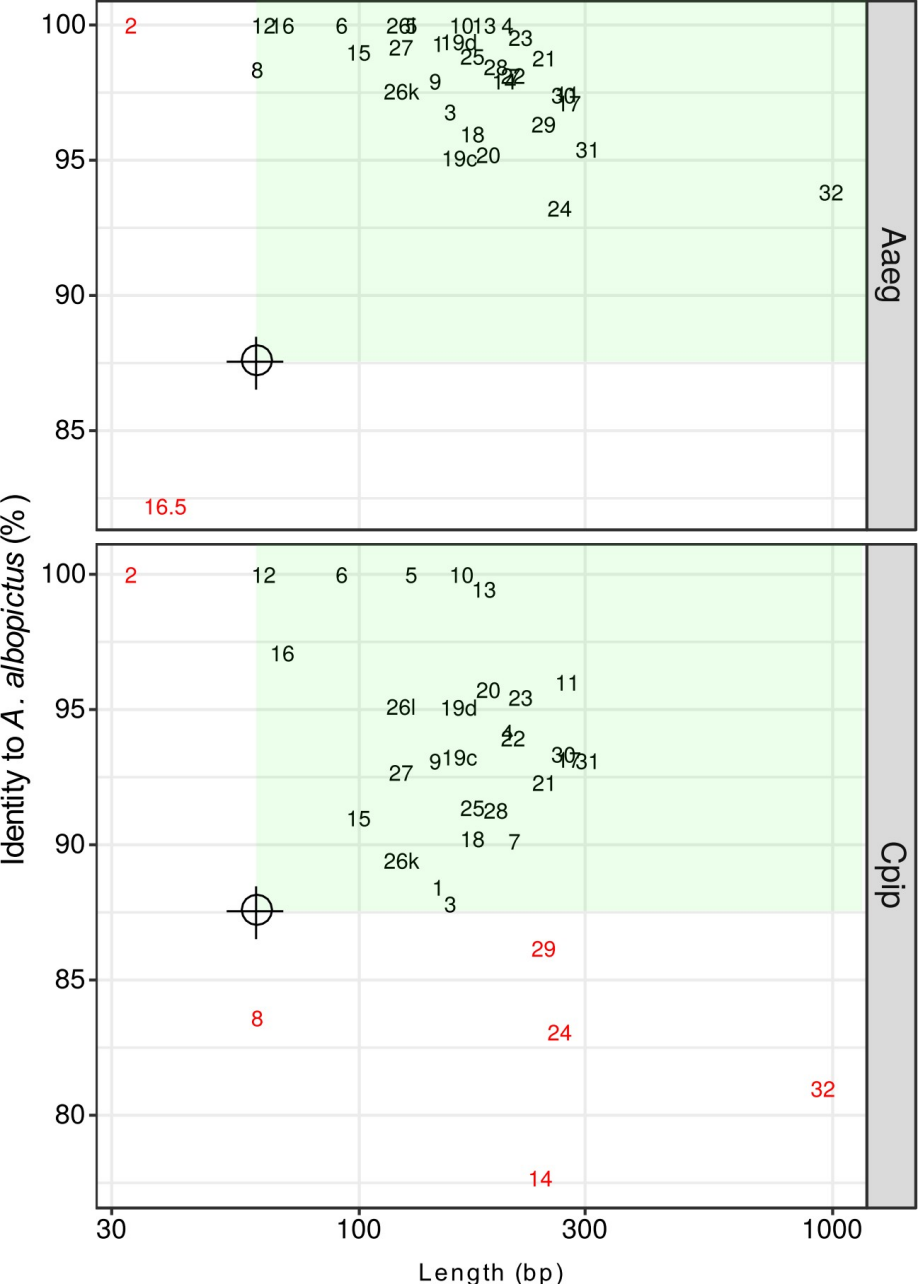
Length and conservation of the *VGSC* exons (CDSs) targeted Length (on a logarithmic scale) and percentage identity to *A*. *albopictus* of each exon in *A*. *aegypti* and *C*. *pipiens* complex. Red exon names are those with low-coverage nucleotides in Fig 3. The green area represents >60 bp length and >87.5% identity.

Even though the probe set was designed on the basis of the *A*. *albopictus* genome sequence only, it successfully enriched *VGSC* CDSs from the gDNA of two other Culicinae mosquito species, *A*. *aegypti* and *C*. *pipiens* complex, which are estimated to have diverged 71.4 and 179 million years ago, respectively, from *A*. *albopictus* [20]. *A*. *aegypti* show almost equal overall enrichment efficiency (relative RPKM of targeted capture sequencing compared to random sequencing) to that in *A*. *albopictus* (Fig 1). On the other hand, the overall enrichment efficiency was lower by approximately 50% in *C*. *pipiens* samples than that in *A*. *albopictus*, though the on-target ratio (absolute RPKM) was still comparable or rather higher in *C*. *pipiens* (Fig 1). This result may be explained by much smaller genome size (579 Mb in the CpipJ2 assembly versus 2.25 Gbp in the C6/36 assembly) of *C*. *pipiens* that might compensate for the lower nucleotide identity to the probes. Applicability of a single probe set to multiple species (e.g., the same genus or family) is obviously advantageous because this obviates the need to prepare each custom probe sets specific for each single species and may enable capture even in species lacking prior genome information. Nonetheless, the evolutionary distance will limit the range of species that one probe set can be applied. In this study, the mapping results on each exon indicated that capture efficiency decreased in some exons (Fig 2). The empirical observation in this study suggests that less than 87.5% in identity or less than 60 bp in size for the homology track of targets could decrease the efficiency of capture significantly (Fig 4). Many exons in *A*. *gambiae* fall short of these criteria (S2 Fig), indicating the current probe set cannot directly be applied to this distant mosquito group. Our probe set especially failed to capture two optionally used exons, 2 and 16.5, in *A*. *aegypti* and *C*. *pipiens* complex, which are among the smallest exons targeted (Fig 2). It is assumed that those tiny exons alone do not provide enough thermostability for probe–target DNA duplex during the capture. Because the probes for those small exons contain flanking intronic sequences of the *A*. *albopictus* genome, those flanking sequences may have provided enough homology region to capture sequences from this species. Although it is straightforward to optimize our probe set further at least for the two other species of mosquito simply by adding species specific probes for those exons and flanking intronic regions, small exons in general will be a major challenge when a probe set is aimed to be used to a wide-range of species because the homology in an intronic region will decay more rapidly than that in an exonic region during speciation. We also missed another exon corresponding to “exon 12” in *Anopheles gambiae* described by Davies et al. [9] during probe design (see Material & Methods). Such tiny and rarely used exons may be difficult to annotate without high-quality high-throughput RNA sequencing data. Nevertheless, in mosquitoes, all such tiny exons are actually situated on the N-terminal intracellular loop or the intracellular loop between domains I and domain II in VGSC, where no resistance-associated mutation has been found so far [8]. Therefore, it is not clear whether ignoring those small exons of *VGSC* from analysis does pose a serious problem for insecticide resistance research.

**Fig 5:**
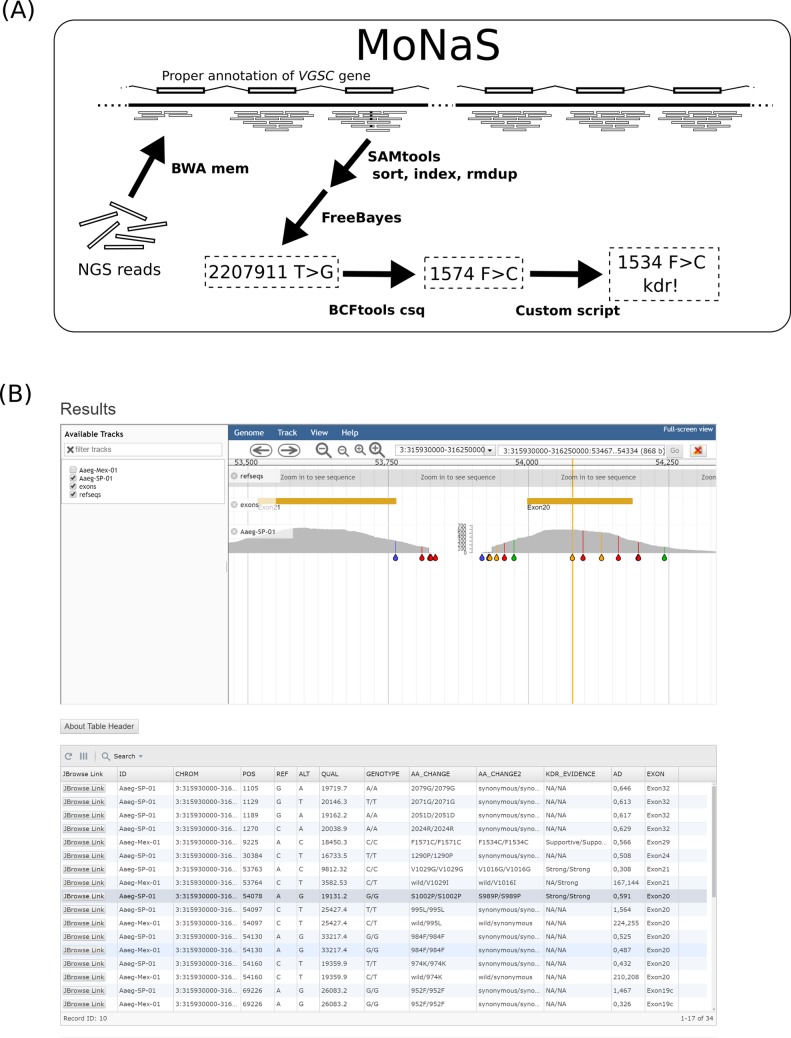
Analytical pipeline MoNaS. (A) A diagram for analytical pipeline MoNaS. MoNaS executes several bioinformatic tools to call variants and aa substitutions. Finally, a custom script converts species aa coordinates to the standard housefly aa coordinates, tells whether each aa substitution is among the known listed *kdr* substitutions and creates a human-readable table from Variant Call Format (VCF). (B) Image of the result output page from MoNaS web-service.

Although our current probe set is designed only from coding exonic regions except for some small exons, hybridization capture methodology allows sequencing flanking intronic region (up to 200 bp, depending on library insert size) in addition to targets [31] (see Fig 5B). Single nucleotide variations (SNVs), which are generally more frequently found in such non-coding regions, provide valuable information for phylogenetic and population genetic information such as origin of resistance allele and their dynamics [17,32].

Copy number variation (CNV) in the *VGSC* gene with potential implication to insecticide resistance have been suggested in *A*. *aegypti* [33]. Although CNV detection was not attempted in this study because no validated CNVs of *VGSC* gene were involved in our samples, the ability of target capture sequencing (or exome sequencing) technology for CNV analysis has been explored in many studies [34,35]. Those approaches rely on differences in sequencing depth caused by copy number change. In this study, median sequencing depth in each exon normalized by total sequencing amount showed relatively uniform distribution among samples when the region was covered by sufficient number of reads (Fig 2). Further normalization could be possible from depth of other gene loci which are considered refractory to copy number change by adding such targets in the probe set, which should be explored in a future study.

The process of genotyping *VGSC* carried out in this study was automated in MoNaS (Fig 5A). This program sequentially runs tools conducting mapping of NGS reads to a reference, sorting, removal of PCR duplicates, indexing for BAM files, variant calling, variant annotation, and finally integration of these results across multiple samples into a single table with conversion of the aa coordinates to those corresponding to the *M*. *domestica* VGSC protein. The automation in MoNaS allows researchers to process raw NGS reads of many samples *via* a simple command line operation without expert knowledge of the bioinformatics field. MoNaS can be run locally (https://github.com/ItokawaK/MoNaS) with appropriate genome reference data. Also, a web-service of MoNaS implemented with JBrowse alignment viewer [36] is provided by NIID Pathogen Genomics Center’s severer (https://gph.niid.go.jp/monas) (Fig 5B).

## Materials and methods

### Design of custom probes

The full-length *VGSC* gene (AALF000723-RA in gene set: AaloF1.2) was found in scaffold JXUM01S000562 in the genome assembly of an *A*. *albopictus* Foshan strain, AaloF1 [20] hosted on vectorbase.org [37]. Because the annotation missed some CDSs (entire exon 19c for instance), we refined the annotation by aligning shotgun-sequenced NGS reads of *VGSC* cDNA [16] using Hisat2 [38] and the *M*. *domestica* VGSC protein sequence (GenBank accession No.: AAB47604) via BLASTX [39]. Compared to AaloF1.2, the refined annotation included three added CDSs, and four extended CDSs (see detail in S1 Table). Among the 35 coding CDSs in total, sizes of 34 (32 + 2 mutually excluding exons) CDSs matched to the *A*. *aegypti VGSC* CDSs annotated by Davies et al. [9]. Therefore, the numbering of exons in this paper was set to be concordant with the *A*. *aegypti VGSC* exons described by Davies et al. [9]. The additional optionally used 45 bp small exon, referred to as exon 16.5 here, was found in cDNA data between exons 16 and 17 in the genome. All CDS sequences, some of which contained a flanking intronic region (for tiny exons less than 120 bp in size) were submitted to the IDT website (https://sg.idtdna.com) to design 120 bp xGen Lockdown biotinylated oligonucleotide DNA probes with 2× Tiling density option. We also included some exons of other genes flanking *VGSC* (AALF020128, AALF020129, AALF020130, AALF000725, AALF000726, AALF000727, AALF000728, and AALF000730) or a gene nested in the intronic region of *VGSC* (AALF020132) during the probe design to take advantage of the population genetic analysis in future studies. From 15 kbp genomic regions in total, 229 probes were designed (S2 Table), of which 145 target *VGSC*. Nonetheless, in the more recent contiguous assembly of the C6/36 cell line (see below), AALF020132, AALF000725, AALF000726, AALF000727, AALF000728, and AALF000730 are not located in the same assembly with the *VGSC* locus. For this reason, in this paper, we evaluate the performance of the probe set only in terms of *VGSC* CDS enrichment.

### Samples

Fifty-six mosquitos either belonging to species *A*. *albopictus*, *A*. *aegypti* or *Culex pipiens* complex—either kept in the laboratory or caught in the wild (Table 1)—served as a source of gDNA. Of those, strains Aalb-SP, Aaeg-SP, and Cpip-JPP were already known to possess haplotypes with 1534C, 989P-1016G, and 1014F aa variants, respectively [16,23,24].

### gDNA extraction

gDNA was individually extracted from the whole body of an adult or pupa using the MagExtractor Genome Kit (TOYOBO). The protocol was modified to conduct the extraction in 8-strip PCR tubes or a 96-well PCR plate as follows. The whole body of a single insect was homogenized in a PCR well containing 50 μl of the Lysis & Binding Solution and zirconia beads (ø 2 mm; Nikkato) in TissueLyser II (QIAGEN) at 25 Hz for 30 s. After that, the samples were centrifuged at 2000 × *g* for 1 min to precipitate large debris, and each supernatant was transferred to a new well containing 50 μl of the Lysis & Binding Solution and 5 μl of DNA-binding Magnetic Beads. The solution was shaken in MicroMixer E-36 (TAITEC) at the maximum speed (2500 rpm) for 10 min, and then, on a magnetic plate, the supernatant was discarded. The beads bound to DNA were washed twice with 100 μl of the Washing Solution and twice with 75% ethanol each. Finally, DNA was eluted with 50 μl of low-TE buffer (0.1 mM EDTA, 10 mM Tris-HCl pH 8.0) by shaking in MicroMixer E-36 at the maximum speed for 10 min. The obtained DNA was quantified with the Qubit Highly Sensitive DNA Assay Kit (Invitrogen). The obtained DNA concentration ranged from 2.3 to 8.6 ng/μl for *A*. *albopictus*, 5.3 to 10 ng/μl for *A*. *aegypti*, and 7.8 to 11 ng/μl for *C*. *pipiens* complex mosquitos.

### Library construction and hybridization capture

Illumina libraries with TruSeq barcode adapters were prepared using NEBNext Ultra II FS DNA Library Prep Kit for Illumina (NEB) on the 1/4 scale of the manufacturer-suggested protocol. Briefly, 4 μl of the gDNA extracted above (without adjusting the concentration) was mixed with 0.4 μl of the Enzyme Mix, 1.4 μl of Reaction Buffer, and 1.2 μl of H_2_O on ice. The mixture was then incubated at 37°C for 10 min followed by incubation at 65°C for 30 min. Those end-prepped DNAs were directly ligated with Illumina adapters by the addition of 0.5 μl of TruSeq 96 dual-index adapters (Illumina) instead of adapters supplied with the kit, 6 μl of the Ligation Master Mix, and 0.2 μl of Ligation Enhancer, with incubation at 20°C for 15 min. Next, the libraries were incubated at 65°C for 30 min to inactivate the ligase; then, all the 56 libraries were pooled together in a single 1.5 ml LoBind tube (Eppendorf). The pooled library was purified with 1.2× SPRIselect (Beckman Coulter) and eluted with 20 μl of low-TE buffer. A 7 μl aliquot of the pooled library was aliquoted and mixed with 0.8 μl of a 10 μg/μl UltraPure Salmon Sperm DNA Solution (Invitrogen) in a PCR-tube. Then, the mixture was concentrated by incubation at 80°C for 10 min while the lid of the tube and thermal cycler were opened. The concentrated library mix was hybridized, captured, and washed with the designed oligo DNA probe set and the xGen Hybridization and Wash Kit (IDT). After that, the streptavidin magnetic beads were subjected to PCR amplification with HiFi Kapa (Kapa Biosystems) for 12 cycles. The amplified library was purified with 1.2× SPRIselect beads and quantified by real-time PCR using P5 and P7 adapter primers and qPCR double quencher probe (6-FAM)-5′-ACACTCTTT-(ZEN)-CCCTACACGACGCTCTTC-3′-(Iowa Black FQ) (IDT) in the PrimeTime Gene Expression Master Mix (IDT). Serial dilutions of the phiX library (Illumina) were used for construction of the standard curve. The quantified library was sequenced on Illumina MiniSeq with the Mid Output Kit (Illumina) for 151 cycles from both ends along with the libraries from other studies.

### Reference genomes and annotation for *VGSC*

Although the probe sets were designed from assembly AaloF1, we chose a C6/36 cell line genome assembly, canu_80X_arrow2.2 [21], as a reference genome of *A*. *albopictus* for further bioinformatic analysis because this assembly has better contiguity and fewer scaffolds than AaloF1 does. In the canu_80X_arrow2.2 assembly, the whole *VGSC* gene was found in scaffolds MNAF02001058.1 and MNAF02001442.1 annotated as Gene IDs LOC109421922 and LOC109432678, respectively, in the NCBI *Aedes albopictus* Annotation Release 101. The two *VGSC* genes were assumed to be redundant haplotigs. To avoid dual mapping of the NGS reads, we purged MNAF02001442 by hard-masking this entire scaffold (replacing all bases with the “N” character) rather than MNAF02001058.1 because LOC109432678 in MNAF02001442.1 has a single frame-shifting nucleotide deletion in the thymine homopolymer track within exon 4 (TTTTTT → TTTTT), which was suspected due to an uncorrected base-calling error. LOC109421922 was defined by the number of transcriptional variants in the NCBI’s annotation because *VGSC* is known to have complex alternative splicing patterns [9]. Nevertheless, we simplified the *VGSC* gene model into two possible transcriptional variants to build a GFF3 annotation file for annotating aa changes. These two transcripts include all the regions of mandatory or optional CDSs but differ by the two mutually exclusive exons “19c/k” and “26d/l,” where one carries exons “19c” and “26k,” and the other contains exons “19d” and “26l.” CDSs of all the transcriptional variants of LOC109421922 were merged via overlaps. Those merged CDSs perfectly matched AaloF1 except for LOC109421922 including exon 16.5 and except for one mutually exclusive exon “26k” whose sequence itself was found to be intact in MNAF02001058.1.

The *VGSC* gene in the chromosome level assembly of the *A*. *aegypti* genome, AaegL5.0 [40], was annotated in the same manner. The whole *VGSC* gene is encoded as AAEL023266 (the NCBI *Aedes aegypti* Annotation Release 101) on chromosome 3. AAEL023266 has 13 transcripts, these CDSs were merged via overlaps as in the canu_80X_arrow2.2 assembly of *A*. *albopictus*. AAEL023266 appeared to be lacking an exon corresponding to exon 16.5, whereas we found its sequence between exons 16 and 17. AAEL023266, however, contains an additional exon between exons 11 and 12. The 21 bp small exon was assumed to correspond to exon “12” in the *Anopheles gambiae* genome described by Davies et al. [9] and is situated within the intracellular loop between domains I and II. We found the sequence homologous to this exon also in the two *A*. *albopictus* genome assemblies, AaloF1 and canu_80X_arrow2.2; which means we had failed to include this exon in the probe design. The complete *VGSC* sequence was also found in scaffold NIGP01000811 and was assumed to be a redundant haplotigs. This scaffold was purged from the assembly by hard-masking.

In the *C*. *quinquefasciatus* genome assembly Cpip_J2 [29], the *VGSC* gene is located in scaffold supercont3.182. The *VGSC* gene in supercont3.182, however, contains a shorter exon 13 which was truncated by scaffolding gap and lacks the entire exon 14. Complete exons 13 and 14 were found in another scaffold, supercont3.1170, which contains an incomplete *VGSC* gene probably as an alternative haplotig. We fused contig AAWU01037504.1 containing exons 13 and 14 of *VGSC* from supercont3.1170 into supercont3.182 to restore the complete coding sequence of the *VGSC* gene, thereby creating supercont3.182_2 (S1A Fig). The *VGSC* in supercont3.182 (and supercont3.182_2) still contained *kdr* aa substitutions, L932F and I936V, as already reported by Davies et al. (2007) plus unusual frameshifting deletions in exons 26l and 32 (S1B Fig). For these reasons, supercont3.182_2 was further polished by the *consensus* module in BCFtools [41] with the “-H 1” option using the variant information for Cpip-JNA-01 in the VCF file generated as described below, thereby finally resulting in supercont3.182_3. The latter scaffold was added to the genome assembly, and the original scaffolds supercont3.182 and supercont3.1170 were purged by hard-masking. We were not able to find an exon corresponding to “exon 16.5” in *A*. *albopictus* and *A*. *aegypti*.

### Bioinformatic analysis

The FASTQ data were mapped to the reference using *BWA mem* (v.0.7.17) [42] with default options. The resultant BAM files were sorted by the *sort* program from the SAMtools suite (v.1.9) [43], and we removed PCR duplicates by the *rmdup* programs from the SAMtools. Variant calling was performed on the resulting BAM files of each species in the FreeBayes software (v.1.2.0) [22] with default options. The variant annotation (for aa changes) was conducted with the *csq* program from the BCFtools suite (v.1.9) [41] with options “*-l -p a*”. Finally, the discovered aa changes were projected onto the corresponding position in the *M*. *domestica* VGSC protein sequence (GenBank accession No.: AAB47604). Those bioinformatic processes (Fig 5A) were automated in pipeline tools *MoNaS* (Mosquito Na^+^ channel mutation Search; https://github.com/ItokawaK/MoNaS) written in the Python3 script language.

For estimating the level of enrichment, five sets of random data on whole-genome shotgun paired-end reads (150 bp × 2, 300 ± 50 bp insert length, 1 million read pairs) from each reference genomic assembly were simulated in the *wgsim* software (https://github.com/lh3/wgsim). The *multicov* program from the Bedtools suite (v.2.27.1) [44] was applied to calculate the number of reads overlapping with any targeted CDS regions. Nucleotide identities of exons were calculated using *Muscle* [45] and BioPython’s *Phylo* package [46]. *R* (v.3.5.1) [47] and the *ggplot2* package [48] were utilized for summarizing and visualizing the data.

### Data accessibility

Raw NGS reads obtained in this study were deposited to DDBJ Sequence Read Archive (BioProjectID: PRJDB7889, see Table 1 for accession no. of each sample). Annotation information for *VGSC* and new reference sequence of *VGSC* gene in *C*. *pipiens* complex used in this study (supercont3.182_3) are provided in S1 Data file. Web service and source codes of MoNaS are hosted in https://gph.niid.go.jp/monas and https://github.com/ItokawaK/MoNaS, respectively.

## Supporting information

S1 Fig Restoring VGSC gene in *C*. *quinquefasciatus* genome (CpipJ2). (A) *VGSC* gene in supercont3.182 lacks entire exon 14 and part of exon 13. Contig AAWU0103754 in the redundant scaffold was merged to supercont3.182 resulting supercont3.182_2 to restore these exons. (B) Exon26l and Exon32 in supercont3.182_2 (supercont3.182_2) each contained three nucleotide deletions each causing frameshift (indicated by arrows). Polishing using NGS reads (from Cpip-JNA-01) corrected these deletions resulting in supercont3.182_3.
(TIF)

S2 Fig Length *Anopheles gambiae VGSC* exons, and nucleotide similarity to the corresponding *Aedes albopictus VGSC* exons. The exon numbering corresponding to those in Davies et al., 2007 (ref. 9 in main text). The green zone represents >60 bp length and >87.5% similarity.
(TIF)

S1 Table Annotation for *VGSC* CDSs used in this study (new) along with annotation in gene set AaloF1.2 (old) in genome assembly AaloF1. The coordinates for start and end are 0-based and 1-based, respectively, as for BED format.
(TSV)

S2 Table List of probe sequences and corresponding genomic interval in AaloF1. The coordinates for start and end are 0-based and 1-based, respectively, as for BED format.
(TSV)

S1 Data Zipped folder containing gff3 annotation files for *VGSC* gene model used in this study and fasta file for the sequence of scaffold supercont3.182_3 of *C*. *pipiens* complex. (ZIP)
