# Supplementary figures and images for: High-throughput genotyping of a full voltage-gated sodium channel gene via genomic DNA using target capture sequencing and analytical pipeline MoNaS to discover novel insecticide resistance mutations
Source: PLoS Negl Trop Dis. 2019 Nov 18;13(11):e0007818. doi: 10.1371/journal.pntd.0007818 (PMC6886866; doi:10.1371/journal.pntd.0007818)

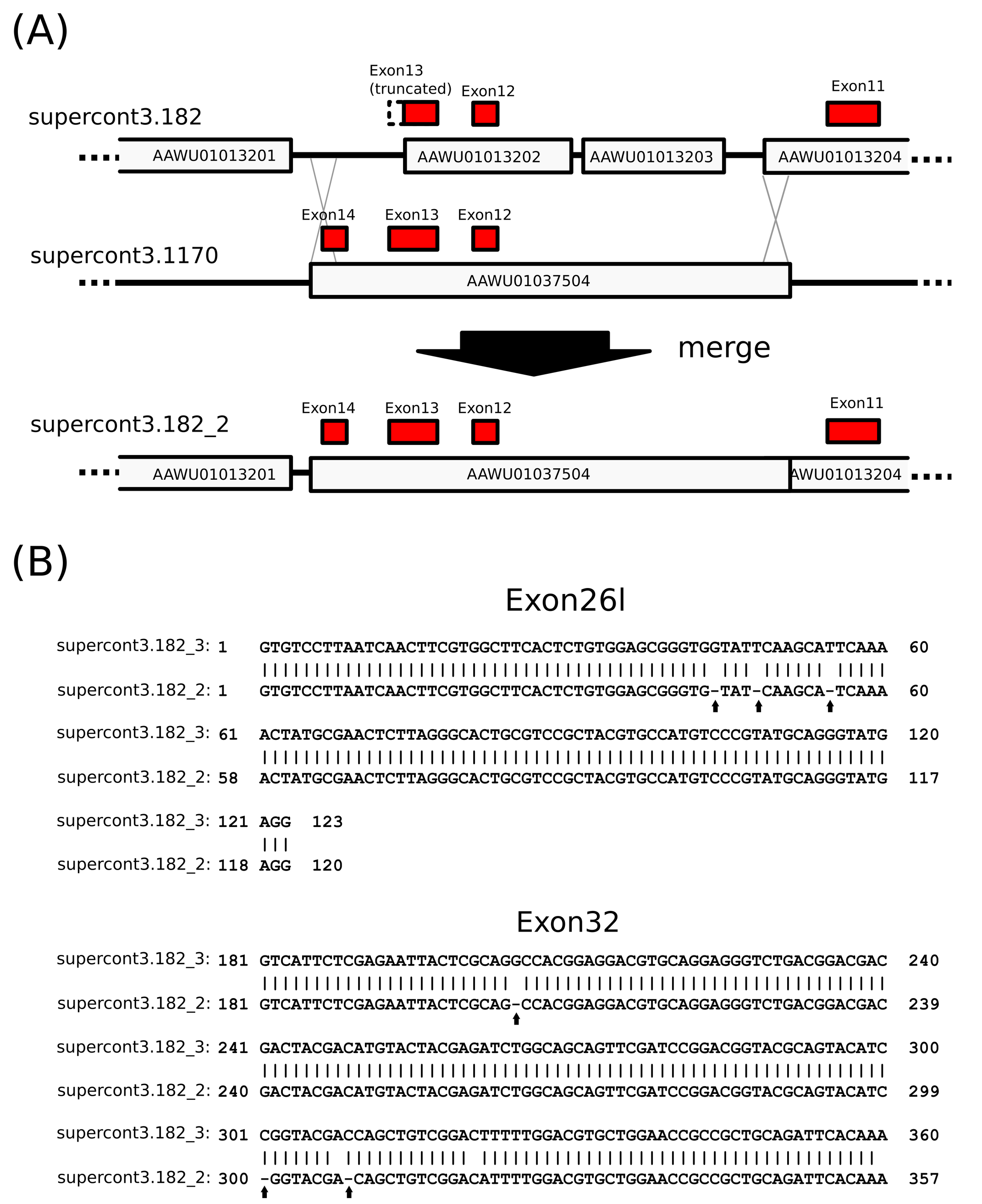

Supplement: S1 Fig — (A) VGSC gene in supercont3.182 lacks entire exon 14 and part of exon 13. Contig AAWU0103754 in the redundant scaffold was merged to supercont3.182 resulting supercont3.182_2 to restore these exons. (B) Exon26l and Exon32 in supercont3.182_2 (supercont3.182_2) each contained three nucleotide deletions each causing frameshift (indicated by arrows). Polishing using NGS reads (from Cpip-JNA-01) corrected these deletions resulting in supercont3.182_3. (TIF) [file pntd.0007818.s001.tif]

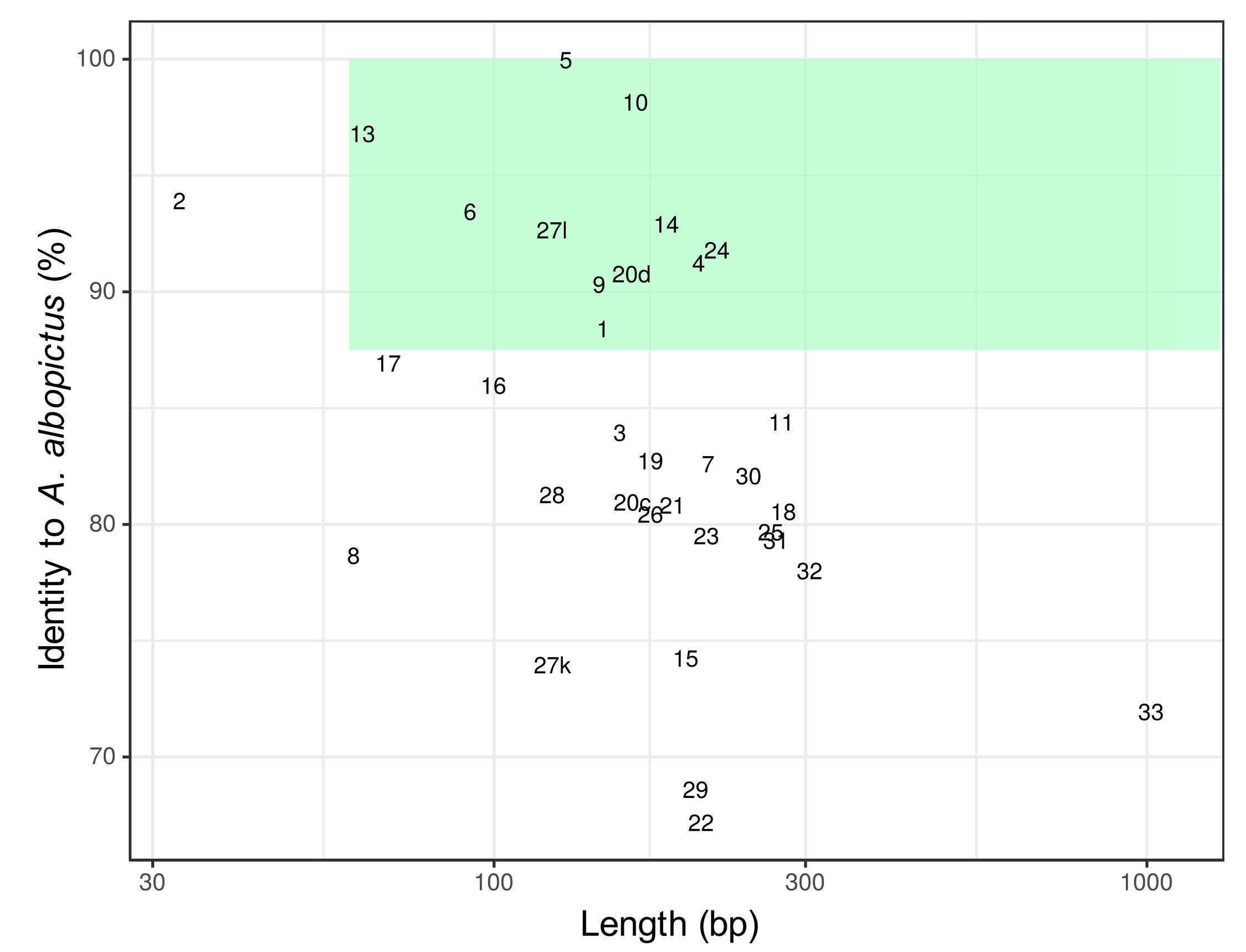

Supplement: S2 Fig — The exon numbering corresponding to those in Davies et al., 2007 (ref. 9 in main text). The green zone represents >60 bp length and >87.5% similarity. (TIF) [file pntd.0007818.s002.tif]
